# 

**Published:** 1887-01-29

**Authors:** 


PRICE ONE PENNY.

No. 18, Vol. I.

January 29th, 1887.

Per Annum, 6s. 6d., post free.

Monthly Parts, 6d.; post free, 7½d.

Ditto per Ann., 7s. 6d., post free.

### THE HOSPITAL

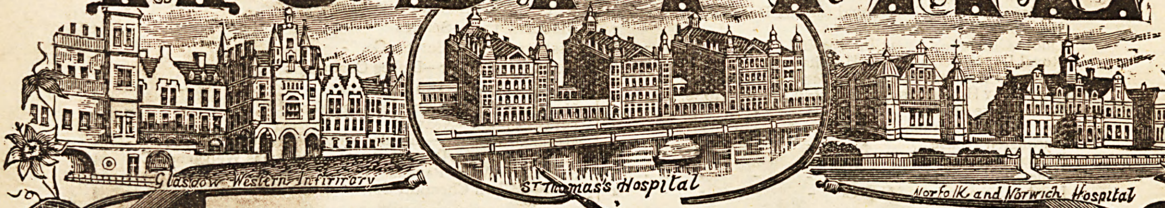

An Institution, Family, and Parochial Journal of Hospitals, Asylums, & all Agencies for the Care of the Sick, Criticism & News.

